# Epidemiological characteristics of imported malaria in Shandong Province, China, from 2012 to 2017

**DOI:** 10.1038/s41598-020-64593-1

**Published:** 2020-05-05

**Authors:** Tao Yu, Yuguang Fu, Xiangli Kong, Xin Liu, Ge Yan, Yongbin Wang

**Affiliations:** Shandong Institute of Parasitic Diseases, Shandong Academy of Medical Sciences, No. 11 Taibai Zhong Road, Jining, 272033 Shandong China

**Keywords:** Psychophysics, Malaria

## Abstract

Shandong Province, China, has been implementing a malaria elimination program. In this study, we analyzed the epidemiological characteristics of malaria imported into Shandong Province between 2012 and 2017 to provide scientific data for the elimination of malaria. In this epidemiological study, we examined the status of malaria in 2012–2017 in Shandong Province, China. Data on all cases of malaria were collected from the online Infection Diseases Monitor Information System to describe and statistically analyze the sources of infection, species of parasite, populations affected, regional distributions, incidence, and temporal distributions of malaria. In total, 1053 cases of malaria were reported in 2012–2017, and all of them were imported. Plasmodium falciparum was the predominant species (77.6%) in Shandong Province; P. vivax malaria accounted for 10.9% of the total number of cases, P. ovale malaria for 2.9%, and P. malariae malaria for 8.2%. Most patients were male (96.8%), most were aged 21–50 years (87.2%), and migrant laborers (77.2%) and workers (6.6%) were at highest risk. The origin of the largest number of imported cases was Africa (93.4%), followed by Asia (5.9%) and Oceania (0.4%). Most cases of imported malaria occurred in June each year and 70% of cases were recorded in six cities during the period of 2012–2017. It is necessary to strengthen malaria surveillance among workers returning home from Africa and Southeast Asia, and to conduct timely blood tests to diagnose and treat imported infections.

## Introduction

Malaria is caused by five species of single-celled eukaryotic *Plasmodium* parasites, *P. falciparum*, *P. ovale*, *P. malariae*, *P. vivax*, and *P. knowlesi*, all of which are transmitted by the bite of malaria-parasite-carrying female *Anopheles* mosquitoes^1,2^. Malaria is a major public health problem of global importance and scientific interest around the world. Although the number of cases of malaria decreased by 25.9% from 2006 to 2016^3^, an estimated 216 million cases^4^ and 719,600 deaths occurred in 2016, and the disease was the sixth leading cause of life lost globally in 2016^3^.

Malaria was widespread in China before 1949, when more than 30 million cases are estimated to have occurred annually, with an annual mortality rate of around 1%^5,6^. In 1955, a national malaria control program was commenced and the disease declined dramatically, with only 2861 cases and no indigenous cases reported in 2017^7,8^. However, malaria remains a serious public health challenge in China, because with growing overseas economic investment, the number of imported cases has increased substantially, and significant numbers of Chinese laborers now work abroad in malaria-endemic countries and then return home^9^.

Shandong Province, which consists of 17 administrative municipalities, is located in Eastern China where malaria is receptive. Only vivax malaria is prevalent in this area, which is home to *A. sinensis*, the only local vector of human malaria. Based on extensive epidemiological surveys of Shandong Province^6,10^, the malaria transmission season lasts from June to November (peaking in August–October). In the early 1960s and 1970s, Shandong Province experienced two large malaria outbreaks, with more than 6 million and 4 million cases of infection annually, respectively^11^. Since then, comprehensive malaria intervention policies and strategies have been adopted, and the number of malaria cases has fallen sharply. By 1988, malaria was almost completely eliminated in Shandong Province, and the number of cases reported annually declined to fewer than 50 in 2000^12,13^. To eliminate indigenous malaria by 2015 and completely eliminate it nationwide by 2020, the National Malaria Elimination Action Plan was implemented in Shandong Province in 2010, following a request by the National Population and Family Planning Commission of the People’s Republic of China^12^. Cases of indigenous malaria started to disappear by 2012^12^. However, the number of cases of imported malaria has increased gradually, with 80 cases reported in 2010 and 97 cases in 2011. Naturally, this situation potentially threatens the success of the malaria-elimination program in Shandong Province, and little is known about the epidemiological characteristics of the imported malarial species. To understand and address this challenge, this study analyzed the epidemiological features of imported malaria in Shandong Province from 2012 to 2017, to provide useful data for the implementation of appropriate strategies and interventions to support the goal of malaria elimination in this region.

## Methodology

**Figure 1:**
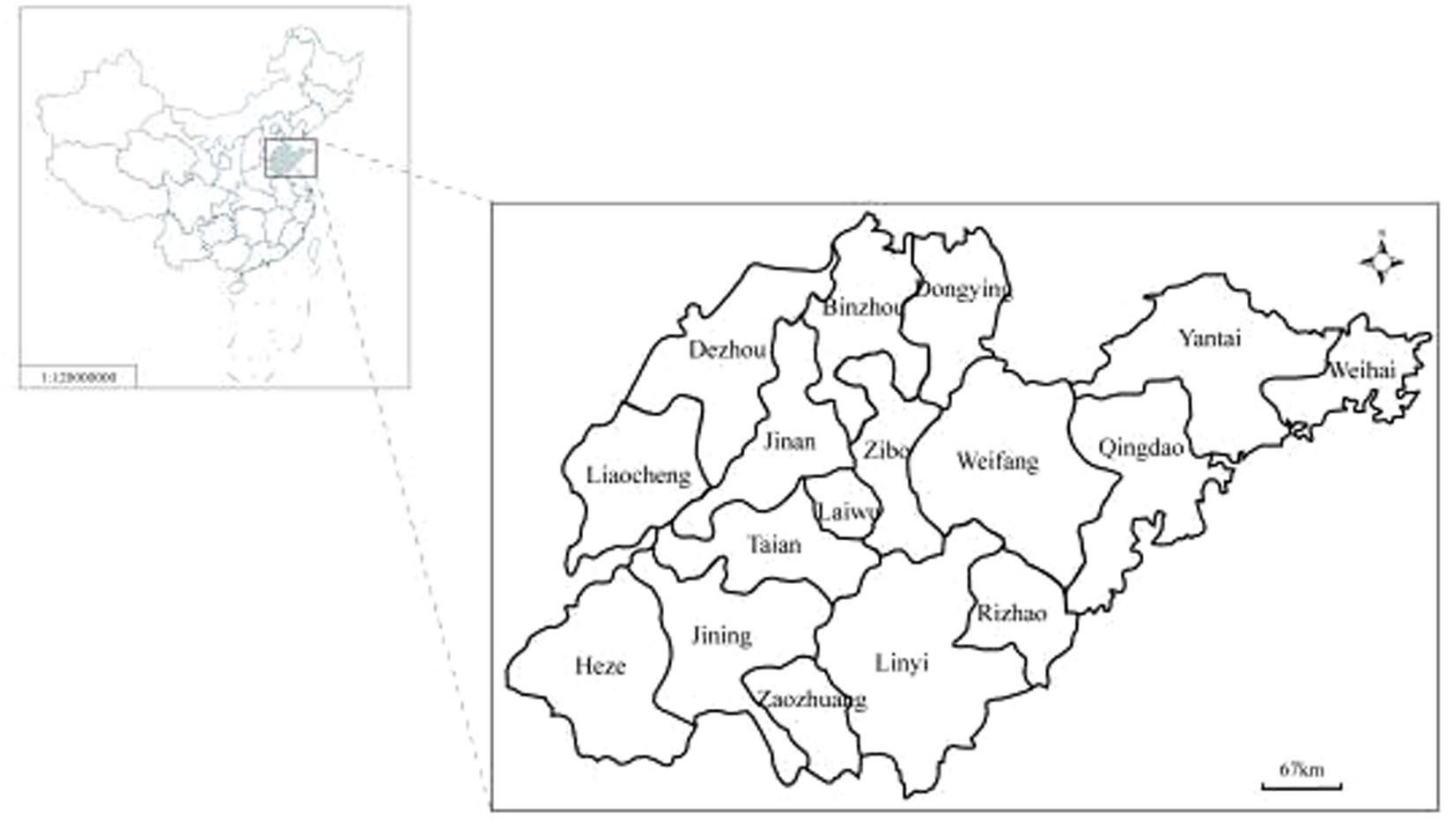
The location of Shandong Province in China.

Shandong Province is situated in east mainland China (34°22.9′–38°24.01′N, 114°47.5′–122°42.3′E) (Fig. 1), adjacent to Hebei, Henan, Anhui, and Jianggsu Provinces. In 2017, Shandong had a population of 100 million, residing in a land area of 157,126 km^2^, and a warm temperate, monsoonal climate, with an annual average temperature of 12 °C and an annual average rainfall of 679.5 mm. The Shandong Provincial Institute of Parasitic Diseases is responsible for the control of malaria in the whole province. The measures taken in this period were the rapid detection and identification of all malarial infections and their appropriate treatment before any secondary infections or local transmission could occur^12^. The program requires that cases of malaria be reported within 1 day and confirmed and investigated within 3 days, and that the appropriate responses to prevent local transmission be instituted within 7 days^14^.

### Case definition

Cases of malaria were defined as follows. (1) ‘Laboratory-confirmed malaria’ refers to patients with a positive result on one type of laboratory test, such as microscopy, rapid diagnostic test, or polymerase chain reaction (PCR). (2) ‘Clinically diagnosed malaria’ refers to patients with malaria-like signs and symptoms but no detectable parasites on a blood examination^15^. Both types of cases were included in this study.

### Case classification

According to the Technical Scheme of Malaria Elimination in China, ‘indigenous malaria’ is defined as malaria in a patients infected within the province in which the diagnosis was made. In contrast, ‘imported malaria’ is defined as malaria whose origin can be traced to an area of transmission outside the province where the diagnosis of malaria was made. The following criteria for imported malaria must be met simultaneously: (1) the patient has received a diagnosis of malaria; (2) the patient has a history of travel to a malaria-endemic area outside China during the malaria transmission season; and (3) the onset time for malaria was <1 month after the patient’s return to China during the local transmission season^16,17^.

### Data collection

Our epidemiological study examined the status of malaria in Shandong Province from 2012 to 2017. Daily disease surveillance data on malaria were obtained from the online Infection Diseases Monitor Information System, which began to cover all Chinese counties in 2008^15^. This information on the cases included sex, age, occupation, residential address, type of disease, date of onset, and date of confirmation.

### Data analysis

The descriptive analysis was conducted with Microsoft Excel and SPSS19 (SPSS, Chicago, IL, USA). The χ^2^ test was used to compare the data counts, and a *P* value of <0.05 was considered statistically significant.

### Ethics approval and consent to participate

The experimental research reported in this study was performed with the approval of the Ethics Committee of the Shandong Provincial Institute of Parasitic Diseases. Human research was performed in compliance with the Declaration of Helsinki and its later amendments. All participants provided their written informed consent to participate in this study.

## Results

### General epidemiological characteristics of malaria

A total of 1053 cases of imported malaria, including five deaths, were reported in Shandong Province from 2012 to 2017, of which 15 were transmitted in other provinces and 1038 were contracted abroad. All cases were diagnosed by the lab-confirmed. From 2010 to 2017, 817 cases of P. falciparum malaria were reported, which accounted for 77.6% of the total number of cases; 115 P. vivax malaria cases accounted for 10.9% of the total number of cases; 31 P. ovale malaria cases accounted for 2.9%; 86 P. malariae cases accounted for 8.2%; and 4 mixed infection malaria cases accounted for 0.4% (Table 1).

**Table 1:** Species type distribution of imported malaria parasites in Shandong Province from 2012 to 2017.

year	*P. falciparum*		*P. vivax*		*P. ovale*		*P. malariae*		Mixed infection		total cases	death cases
	cases	(%)	cases	(%)	cases	(%)	cases	(%)	cases	(%)		
2012	67	72.04	21	22.58	0	0.00	3	3.23	2	2.15	93	0
2013	116	88.55	7	5.34	4	3.05	4	3.05	0	0.00	131	0
2014	121	80.67	16	10.67	4	267	9	6.00	0	0.00	150	3
2015	164	77.36	21	9.91	9	4.25	18	8.49	0	0	212	2
2016	196	76.56	28	10.94	8	3.12	22	8.59	2	0.78	256	0
2017	153	72.51	22	10.43	6	2.84	30	14.22	0	0	211	0
Total	817	77.59	115	10.92	31	2.94	86	8.17	4	0.38	1053	5

**Figure 2:**
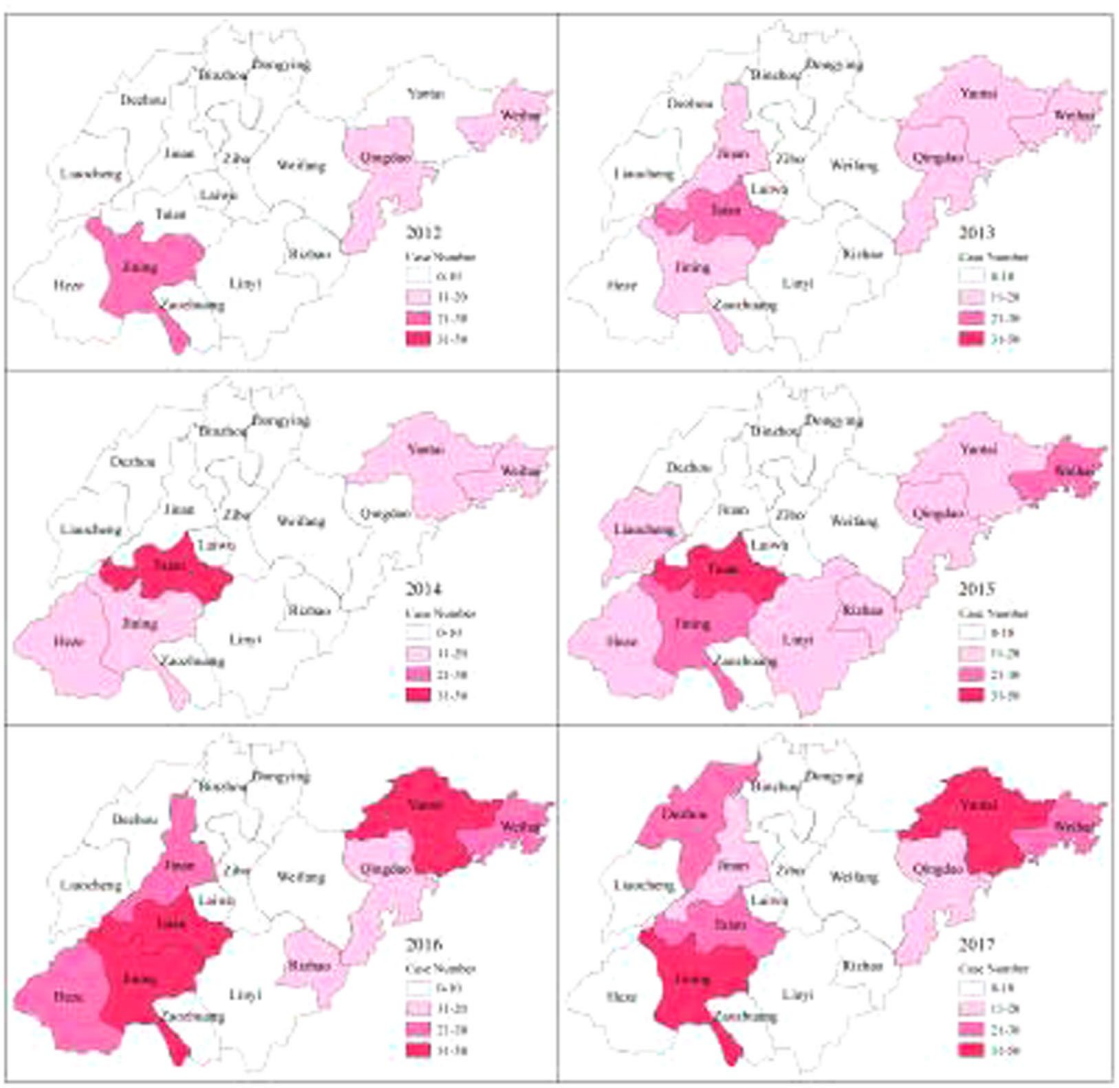
Geographical distribution of malaria cases in Shandong province from 2012 to 2017.

Cases of imported malaria were recorded in all 17 cities of Shandong Province in 2012–2017 (Fig. 2). However, this 6-year analysis showed that most of these cases were recorded in the cities of Taian, Jining, Yantai, Weihai, Qingdao, and Jinan, which reported 182, 146, 123, 122 89, and 82 cases of malaria, respectively, accounting for 70.7% of the total cases (Table 2).

**Table 2:** Number of malaria cases in Shandong Province from 2012 to 2017, by city [n].

Cities	2012	2013	2014	2015	2016	2017	Total
Jining	30	14	14	25	31	32	146
Jinan	10	15	9	7	24	17	82
Weihai	14	19	13	25	23	28	122
Yantai	6	19	12	20	33	33	123
Taian	7	29	40	44	41	21	182
Qingdao	11	12	10	16	20	20	89
Rizhao	6	9	3	12	14	9	53
Heze	1	3	11	15	22	2	54
Weifang	2	4	8	3	3	7	27
Zibo	2	0	6	2	9	4	23
Zaozhuang	0	2	6	2	8	1	19
Dezhou	1	0	9	1	9	22	42
Laiwu	1	4	2	2	4	1	14
Linyi	1	0	5	18	9	10	43
Liaocheng	1	1	0	16	0	2	20
Bingzhou	0	0	1	2	5	0	8
Dongying	0	0	1	2	1	2	6
Total	93	131	150	212	256	211	1053

From 2012 to 2017, 1035 male and 18 female patients were diagnosed with malaria, accounting for 96.8% and 3.2% of the total cases, respectively, and the male-to-female ratio was 57.5:1 (1035:18), with similar distributions across all age groups (χ^2^ = 8.39, *P* > 0.05) (Table 3). Nine hundred nineteen (87.3%) cases occurred in people aged 21–50 years, of which 97.9% were male and 2.1% female. Only two children were infected among all the cases. Of all the cases, 77.2% (813/1053) involved migrant laborers, 6.6% (69/1053) were workers, 5.5% (58/1053) were businessmen, and 10.7% (113/1053) involved other professions (e.g., fishermen, teachers, drivers, and translators) (Table 3).

**Table 3:** Demographic characteristics of malaria cases in Shandong Province, 2010–2017.

Variables	2012	2013	2014	2015	2016	2017	Total
Male	92	130	138	212	255	208	1035
Female	1	1	12	0	1	3	18
≤20	1	1	1	1	2	0	6
21–30	18	18	23	52	51	31	193
31–40	43	53	68	59	99	84	406
41–50	26	52	45	96	98	90	407
≥51	5	7	13	4	6	6	41
Businessmen	8	10	10	13	12	5	58
Workers	6	8	8	14	9	24	69
Overseas laborers	69	98	107	155	216	168	813
Farmers	0	2	0	3	2	1	8
Students	1	0	1	1	4	0	7
Children	0	1	0	0	1	0	2
Others*	9	12	24	26	12	13	96
Total	93	131	150	212	256	211	1053

*Others include fishermen, teachers, drivers, translators, for example.

**Figure 3:**
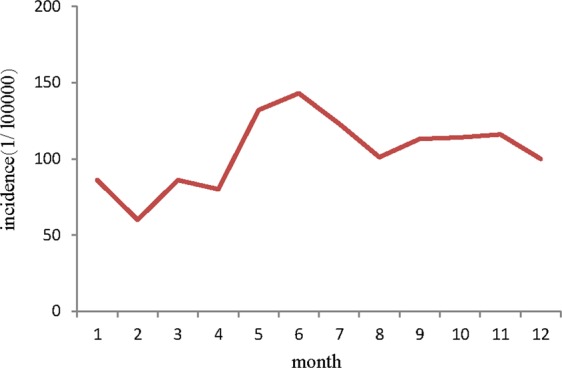
Monthly distribution of imported malaria in Shandong Province from 2012 to 2017.

In terms of the seasonal distribution of imported malaria in 2012–2017, cases were reported in every month, but weak peaks were observed in May–July. The highest proportion of cases of imported malaria occurred in June each year in the period 2012–2017, when 117 malaria cases accounted for 11.1% of the total number of cases. The lowest monthly incidence (5.7%) of malaria occurred in February during 2012–2017 (Fig. 3). However, the numbers of cases that occurred in the different months were similar in 2012–2017 (χ^2^ = 8.375, *P* > 0.05).

The sources of the 1053 cases of imported malaria were countries outside China. Of these 1053 foreign cases, 984 were from 18 countries and regions in Africa (accounting for 93.4% of the total foreign imported cases), 62 were from eight countries and regions in Asia (accounting for 5.9% of the total foreign imported cases), and only six cases were from countries on other continents (Table 4). Equatorial Guinea, Angola, and Nigeria were the dominant sources of imported malaria, accounting for 20.76%, 15.40%, and 10.26% of the total cases, respectively.

**Table 4:** Reported source of imported malaria in Shandong Province, 2012–2017.

Country		Plasmodium species					Total
		*Plasmodium falciparum*	*Plasmodium vivax*	*Plasmodium ovale*	*Plasmodium malariae*	Mixed infection	
Africa	Equatorial Guinea	202	21	5	16	0	984
	Angola	148	15	6	8	0	
	Nigeria	99	6	2	10	0	
	Ghana	72	5	2	9	1	
	Libya	62	6	2	5	0	
	Congo	57	6	3	3	0	
	Guinea	23	4	1	5	0	
	Tanzania	23	3	1	2	1	
	Cameroon	13	4	1	2	0	
	Sudan	19	5	1	5	0	
	Other African countries	84	6	4	6	0	
Asia	Palestine	3	9	0	3	0	62
	Indonesia	3	11	0	2	0	
	Myanmar	3	4	0	3	0	
	India	1	2	0	1	1	
	Cambodia	2	1	0	3	0	
	Bhutan	1	2	0	1	1	
	Other Asian countries	2	1	0	2	0	
Oceania	Papua New Guinea	0	3	1	0	0	4
South America	Venezuela	0	1	2	0	0	3
Total		817	115	31	86	4	1053

## Discussion

In this study, the longitudinal surveillance data of imported malaria in Shandong Province, China from 2012 to 2017 was analyzed to identify the epidemiological characteristics. Four main epidemiological characteristics were observed. Firstly, the most cases of imported malaria were concentrated in the cities of Taian, Jining, Weihai, Yantai, Qingdao, and Heze. Secondly, most cases of imported malaria were migrant workers and mostly were adult men aged 21–50 years. Thirdly, no seasonal differences were observed in the imported malaria cases. Fourthly, falciparum malaria was dominant species among the cases of imported malaria and most of these cases were originatein Africa. The pattern of imported malaria was associated with the transmission intensity in the locations visited by the patients, the number of travelers to each endemic area, the demographic and ethnic backgrounds of the travelers, the activities undertaken during their trips, the type of accommodation they used, and their adherence to prophylaxis^17^.

Historically, *P. vivax* is the only malarial parasite transmitted by *A. sinensis*, and this parasite caused a very serious endemic in Shandong Province. The prevalence of malaria has declined significantly with the implementation of high-level coverage of antimalarial interventions in this province, including mass drug administration, the distribution of insecticide-treated mosquito nets, and indoor insecticide spraying^6^ and the province has been in the malaria-elimination phase of its control program since 1988. Consequently, the number of cases of locally transmitted malaria declined dramatically, and disappeared after 2012. The absence of local malaria was achieved by implementing the national malaria elimination program and by continuous investments from international and domestic funding bodies to support the diagnosis and treatment of malaria, indoor residual mosquito spraying, and the distribution of insecticidal bed nets^18^. Another factor that supported the control of malaria was the cooperation between Shandong and four other provinces (Jiangsu, Hubei, Henan, and Anhui) since 1974, which also contributed to the elimination of local malaria. The cooperative malaria control strategies included the application of unified measures, the regular exchange of experience and information, and the annual inspection and monitoring of malaria^19^.

As seen in other studies^16,20^, imported malaria accounted for most cases in this study and *P. falciparum* was the predominant species in these cases, during the elimination stage of the program. The majority of imported cases in Shandong Province originated in African countries where malaria is endemic and *P. falciparum* is the main parasitic species in humans^21,22^. However, because *P. vivax* is the only epidemic malarial species occurring in humans in Shandong Province, the medical staff in this province lack the knowledge required to prevent and treat falciparum malaria. Therefore, the education and training of staff must be strengthened in this area.

During the malaria-elimination stage in the control program, imported malaria has become a key public health challenge in Shandong Province and heightened the status of malaria in our country^18^. The elimination strategy at this stage of the program is mainly concerned with the detection, investigation, and reduction of malaria cases and their foci. The ‘1–3–7’ strategy has been implemented to ensure the effective detection,confirmation of cases and proper case classification and treatment, which is specifically focusing on investigation and action^14^ to ensure that the cases of imported malaria are not transmitted to local people. Strengthening the prevention, detection, and management of imported malaria requires the cooperation of public health officials, healthcare workers, immigration and quarantine services, the commercial sector, and the police^22^.

It has been reported that severe cases of malaria have been associated with the failure of medical staff to recognize the disease in its early stage^23^. Therefore, the early recognition, diagnosis, and treatment of imported malaria should be prioritized^24^, and healthcare workers should be aware of the need for the rapid diagnosis and treatment of imported cases, particularly those returning in Africa. However, healthcare workers lack the skills required to detect imported malaria and require better training on the current situation of malaria in China^25^.

Although imported malaria was reported the whole territory of Shandong Province between 2010 and 2017, most cases were reported in the several cities. This is consistent with other reports that although cases of imported malaria are scattered across various regions, they are also relatively concentrated in some areas^13^. The high frequency of travel and trade in these cities may be a contributing factor^26^. This is also consistent with the fact that the incidence of rural migrant workers is significantly higher in these areas than in other areas. One study reported that the proportion of businessmen infected with malarial parasites was higher among imported cases^26^, suggesting that as the mobility of the population increases in concert with increased economic development, we must strengthen the monitoring of this section of the mobile population.

Several epidemiological studies have reported that males and people aged 21–50 years were predominant among the cases of imported malaria^13,26,27^, and the findings of our study support this finding. Specifically, our analysis of the age, sex, and occupation distributions showed that males aged 21–50 years were the predominantly affected group in Shandong Province in 2012–2017. A possible explanation for the large number of nonimmune male adults in the 21–50 age is that they travel or migrate to malaria-endemic areas for work more likely. In Shandong Province, rural migrant workers accountted for a large proportion of overseas laborers. Many overseas laborers work in high-risk environments, such as natural forests, palm oil or rubber plantations, and fish farms^28^, and lack any awareness of the risk of malaria and the personal protection required against it^29^. This increases the risk of infection with malarial parasites. To improve their knowledge of the prevention and treatment of malaria, occupational protection measures should be implemented and strengthened to reduce the risk of malarial infection among overseas laborers^30^.

Lai *et al*. reported that small peaks of imported malaria occurred in January and February in China^31^. A possible reason for this is that overseas laborers return to China to celebrate the Chinese New Year holiday in these months. However, our analysis of the seasonal fluctuations in imported malaria did not include these two weak peaks, but only a single peak in May–July observed. This peak may be owing to the return to China of workers from overseas countries to engage in agricultural work during May–September^32,33^. Although the distribution of imported malaria fluctuated seasonally, the number of cases was similar in every month. This suggests that the medical staff not only attended returning workers in the peak period but also in other months.

The current results suggest that the “1–3–7” malaria epidemic management model needs to be rigidly followed in Shandong Province to enhance the management of the epidemic and the investigation and eradication strategies associated with the epidemic needs to be standardized, such as enhancing health education before people leave the region and border screening for malaria when people enter the region. Additionally, the early exact detection of malaria and opportunely clinical treatment play a pivotal role in decreasing malaria morbidity and mortality, postponing Chem Resistant and improving malaria control, so it is important to ensure prevailing diagnostic detecting procedures for all doubted malaria cases.

Our study also had several limitations. Firstly, the number of patients diagnosed with malaria may have been underestimated because people with malaria may not always seek medical treatment. Secondly, cases of imported malaria may have been misdiagnosed in malaria-free or hard-to-reach areas, even though individual Chinese case-based malaria surveillance systems operated well during the malaria-elimination stage of the control program^18^. Thirdly, because the reporting system was based on passive surveillance, the quality of the reported data may have been affected by the detection capacity, the reporting methodology, and/or the accessibility and availability of healthcare facilities.

## Conclusions

In summary, imported malaria has become a challenge to malaria elimination in Shandong Province, China. An effective malaria management and control system for the migrant population is a necessary strategy for detecting cases of imported malaria. The Ministry of Tourism, labor service companies, entry–exit inspectors, and the Department of Health must cooperate to strengthen malaria monitoring and the screening of laborers and travelers arriving from malaria-endemic areas. Medical laboratory staff should also receive better training in the diagnosis and detection of imported malaria. Finally, better information on malaria prevention and treatment should be provided to people in China who visit malaria-endemic regions of the world.

## Data Availability

All data relevant to this manuscript have been stored in a separate file, which is freely available to external investigators upon request.
